# A Four-Step Cascade Drug-Release Management Strategy for Transcatheter Arterial Chemoembolization (TACE) Therapeutic Applications

**DOI:** 10.3390/polym13213701

**Published:** 2021-10-27

**Authors:** Ying-Jiun Hsieh, Hung-Wei Cheng, Hung-Yu Chen, Ming-Wei Lee

**Affiliations:** 1 Graduate Institute of Technology Management, National Chung Hsing University, Taichung City 402, Taiwan; arborfish@dragon.nchu.edu.tw; 2 Department of Materials Science and Engineering, National Chiao Tung University, Hsinchu 30010, Taiwan; mlb14756@gmail.com; 3 Department of Clinical Laboratory, Chung Shan Medical University Hospital, Taichung 40201, Taiwan; c135791010@gmail.com; 4 Department of Speech and Language Pathology and Audiology, Chung Shan Medical University, Taichung 40201, Taiwan

**Keywords:** cascade antitumor drug delivery, gellan gum polysaccharide, graphene nanoparticles, transcatheter arterial chemoembolization

## Abstract

The purpose of this study was to develop a four-step cascade drug-release system for transcatheter arterial chemoembolization (TACE) therapeutic applications according to disease-driven and patient-focused design theories. The four steps underlying these strategies involve the blockage of nutrient supply, nanoparticles, codelivery and the cell cytotoxic effect. Calibrated spherical gellan gum (GG) and nanoparticle-containing gellan gum microspheres were prepared using a water-in-oil emulsification method. Self-assembled nanoparticles featuring amine-functionalized graphene oxide (AFGO) as the doxorubicin (Dox) carrier were prepared. The results confirm that, as a drug carrier, AFGO–Dox nanoparticles can facilitate the transport of doxorubicin into HepG2 liver cancer cells. Subsequently, AFGO–Dox was introduced into gellan gum (GG) microspheres, thus forming GG/AFGO–Dox microspheres with a mean size of 200–700 μm. After a drug release experiment lasting 28 days, the amount of doxorubicin released from 674 and 226 μm GG/AFGO–Dox microspheres was 2.31 and 1.18 μg/mg, respectively. GG/AFGO–Dox microspheres were applied in a rabbit ear embolization model, where ischemic necrosis was visible on the ear after 12 days. Our aim for the future is to provide better embolization agents for transcatheter arterial chemoembolization (TACE) using this device.

## 1. Introduction

The development of drug delivery systems for disease management at the laboratory stage is often referred to as the initial laboratory phase. The idea behind this is to develop a rational model for drug delivery systems that can be used by stakeholders as a tool to support technology innovation. Barry, S.-T. et al. (2017) [1,2], described the concept of using disease-driven and patient-focused design theories in anticancer strategy development from an industry perspective. In our study, we applied these design theories to develop a drug-release management strategy for hepatocellular carcinoma (HCC) therapy. Disease-driven design is rooted in understanding the implications of biology for cell medicine behavior in order to select a carrier that is able to exploit pathophysiology. The approaches outlined in this article focus on the development of drug delivery strategies for hepatocellular carcinoma therapy and were designed to overcome the limitations of tumor biology by increasing penetration and delivering more drug away from the vasculature, in addition to prolonging retention of high concentrations of drug in the tumor, thereby increasing drug availability. In patient-focused design theory, understanding the off-target effects of cancer drugs is as important as evaluating their efficacy. An off-target effect is the biological activity of a drug acting on targets other the intended biological target. It most commonly contributes to side effects [3]. Thus, reducing the side effects of drugs and enhancing safety represent the core of this design. Drugs are delivered to a specific tissue through a delivery system, and drug accumulation is promoted to improve the therapeutic effect as a function of the relative clearance rate of the drug by the tumor and normal tissue [4]. By improving the tolerance of the patient to the drug and reducing its toxicity, the patient can eventually receive a higher lifetime dose. Drug carrier development strategies are based on both theories, i.e., disease-driven design and patient-focused design. A development strategy that is based on the disease-driven design theory usually involves changing the material, structure (such as aperture or size), and surface characteristics of the drug delivery carrier. In order to establish the safety, tolerability and durability of a drug for patients, a development strategy that is based on patient-focused design theory will involve multifunctional carriers and combination therapies as the core of the development. A drug is composed of two parts: the main body (with a pharmaco-logical composition) and the delivery carrier. The drug delivery carrier is an auxiliary component; however, it represents the key target of strategies that are based on the theory of patient-focused design. This allows any systemic adverse reactions that occur during the process of drug administration to be relieved and enables patients to receive higher lifetime doses to cope with malignant (cancer) and chronic diseases.

Accordingly, this study was conducted to design a four-step cascade drug-release management strategy for the treatment of liver cancer patients. The four steps were interrelated, and each had its own important strategy to address the goal of cancer treatment. Each step corresponds to a cancer treatment strategy [5]. The first step was based on nutritional diet strategies, whereby vascular embolization particles were de-signed to block nutrient supply and thereby achieve the purpose of killing cancer cells. According to the concept of patient-focused design, vascular embolism is a local treatment that has fewer side effects than chemotherapy. The second and third steps adopted both a nanoparticle strategy and codelivery strategy, combining graphene nanoparticles with the chemotherapy drug doxorubicin (Dox) [6]. Nanoparticles were used as the drug carrier, as they can prolong the retention time of the drug in the blood as well as reduce opsonin absorption and reticuloendothelial system (RES) recognition [7]. The codelivery strategy allows the cancer cells to increase their uptake of drug in addition to increasing drug penetration into cells [8]. The fourth step involves a cytotoxic strategy, whereby graphene and Dox are transported to the cell and are subsequently separated in response to a pH-sensitive stimulus. Because the main function of Dox is to kill cancer cells, its concentration is a key factor in cancer treatment. The ideal drug carrier enables high bodily availability with a low dose of drug, thereby allowing the patient to receive a higher lifetime dose to fight the disease.

Embolization blocks the blood supply to a tumor, leading to tumor cell death. To enhance their therapeutic effect, chemotherapy drugs are loaded with an embolic agent, a treatment referred to as chemoembolization [9]. Transcatheter arterial chemoembolization (TACE), which is the recommended treatment for mid-stage hepatocellular carcinoma (HCC), allows the drug to be delivered to a localized tumor site and selectively blocks the blood supply to the tumor [10]. TACE has less of an effect on normal cells, since approximately 75% of the liver’s blood supply comes from the hepatic portal vein. Compared to traditional chemotherapy, TACE can control drug release to reduce systemic toxicity and extend its duration at the tumor site, thereby inducing tumor necrosis without an adverse reaction [11,12]. Moreover, TACE can induce tumor-associated antigens (TAAs) to activate immune components, such as Th17, CD4 and CD8 T cells [13]. However, some side effects may occur, including pulmonary thromboembolisms, liver abscesses, and bile duct lesions [14]. Embolization reagents are typically designed to achieve good TACE efficacy. There are some requirements for ideal TACE reagents, as follows: (1) quick blockage of the blood flow following intra-arterial injection; (2) anticancer drug release for localized treatment; (3) suitable degradation time to prevent thrombi. Current TACE agents (such as microspheres, beads and hydrogels) have been developed using polymers [15].

Conventional TACE (cTACE) and TACE with drug-eluting beads (DEB-TACE) are two types of TACE agents that are currently available [16]. cTACE, which involves a mixture of anticancer drugs and lipiodol and embolization reagents (e.g., gelatin microspheres), is commonly used for HCC treatment, although it can induce serious systemic toxicity and hepatic artery injury [17]. On the other hand, DEB-TACE (e.g., DC bead^®^) is a drug delivery system (DDS) for microsphere embolic agents that can successfully increase local drug concentration, leading to tumor necrosis and slow anti-cancer drug release. This reduces the side effects that are caused by cTACE, because chemotherapeutic drugs (e.g., doxorubicin (Dox) and epirubicin) can be loaded into DC beads with sulfonate groups to control release as a function of ionic changes [18]. In addition, the concentration of Dox can be effectively increased, thereby enhancing the therapeutic effect, as shown in a liver tumor rabbit model. One study reported that smaller microspheres (<100 µm) can provide more effective embolization than larger microspheres because they can enter the thin branches of blood vessels near a tumor site. However, DC beads^®^ cause irreversible embolism at the targeted site, and their main material is based on polyvinyl alcohol (PVA), which induces calcification and inflammation [19]. Therefore, the aim of this study was to clinically develop an ideal DDS with suitable microspheres (in terms of material and size) for TACE.

Gellan gum (GG) is a linear, anionic extracellular polysaccharide with repeating tetrasaccharide units of D-glucose, D-glucuronic acid, D-glucose and L-rhamnose sourced from Pseudomonas elodea. Gellan gum is a food additive that functions as a stabilizer, thickening agent and a versatile structuring and gelling agent in a wide variety of foods. Gellan gum has been investigated as a candidate material for biomedical engineering because of its biocompatibility and low cytotoxicity. In our previous studies, we verified the chemical and biological characteristics of gellan gum and identified it as a suitable starting point for developing implantable biomedical devices [20,21]. In the present experiment, we adopted gellan gum as the substrate for developing a microspherical embolization agent. Gellan gum was formed into microspheres via emulsification [22]. In this study, we developed long-term, biodegradable microspheres that encapsulate nanosized graphene carrying chemotherapy drugs to embolize the hepatic artery and achieve dual release. Here, gellan gum (GG) was used as the main material of the microsphere due to its biocompatibility as well as its proven suitability as an embolic agent in terms of slow swelling and degradation [23,24]. Moreover, the application of amine-functionalized graphene oxide (AFGO) improved the reaction with polymers and drugs [25,26]. Doxorubicin (Dox) is the main chemotherapeutic drug currently used for liver cancer treatment. The microsphere-encapsulated nanoparticles were synthesized via emulsion to form a GG/AFGO–Dox dual-release system. Its successful generation was confirmed by systemic analysis of its morphology, drug release kinetics, and in vitro and in vivo efficacy in terms of embolization.

## 2. Materials and Methods

### 2.1. Materials

Gelzan™ CM, calcium chloride, mineral oil, doxorubicin hydrochloride (Dox-HCl) and Span^®^ 85 were purchased from Sigma-Aldrich (St. Louis, MO, USA). Gibco™ DMEM, Gib-co™ Fetal Bovine Serum (FBS), Gibco™ Penicillin-Streptomycin, Invitrogen™ Presto-Blue™ Cell Viability Reagent and Gibco™ Trypsin-EDTA (0.05%) were purchased from Thermo Fisher Scientific (Branchburg, NJ, USA). FITC Annexin V Apoptosis Detection Kit I was purchased from BD Pharmingen™ (San Diego, CA, USA). Sodium Nitrate, hydrogen peroxide and Sodium acetate were purchased from HAYASHI PURE CHEMICAL IND. (Osaka, Japan), Ltd. (HPC). Sulfuric Acid was purchased from UNION CHEMICAL WORKS Ltd. (Hsinchu, Taiwan). Potassium permanganate and ferric chloride were purchased from SHIMAKYU’S PURE CHEMICALS (Osaka, Japan). Ethylenediamine was purchased from ACROS Organics™ (Carlsbad, CA, USA). Ethylene glycol was purchased from PanReac AppliChem (Chicago, IL, USA). Graphite powder was donated from the Department of Public Health (Taichung, Taiwan), Chung Shan Medical University. All the samples in this study were sterilized in 70% (*v*/*v*) ethanol solution at 25 °C for 24 h. Next, samples were washed three times in sterile phosphate buffered saline (PBS) for five minutes each time. Finally, samples were dried or used directly for testing.

### 2.2. Amine-Functionalized Graphene Oxide (AFGO) Synthesis

Amine-functionalized graphene oxide (AFGO) was obtained from graphene oxide (GO). GO was prepared by Hammer’s method. Briefly, 4 g graphite powder was mixed with 2 g NaNO_3_ and 92 mL H_2_SO_4_ and then the mixture was stirred at 4 °C for 5 min. Next, 12 g KMnO_4_ was added at room temperature and stirred for 5 min and then heated to 35 °C for 30 min. Then, 184 mL deionized water was added in the mixture at 65–75 °C for 15 min. Finally, hydrogen peroxide solution (H_2_O_2_, 3% in water) was added for 1 h. After the mixture was centrifuged at 2000× *g* for 10 min to remove the excess H_2_O_2_ and washed 3 times with ddH_2_O, it was dried at 60 °C for 4 days. The 0.5 g GO was mixed with 1 g Iron (III) chloride (FeCl_3_), 3 g sodium acetate (C_2_H_3_NaO_2_), 20 mL ethylene glycol (C_2_H_6_O_2_) and 10 mL ethylenediamine (C_2_H_8_N_2_) in an autoclave at 200 °C for 2 h. The mixture was washed with deionized water at 2000× *g* for 10 min and it was dried at 60 °C for 1 day.

### 2.3. Preparation of Gellan Gum (GG), GG/AFGO and GG/AFGO–Dox Microsphere

The gellan gum (GG) microsphere was prepared by an emulsification and cation-induced cross-linking process [22]. The aqueous GG (0.3% *w*/*v*) solution was stirred for 15 min at 85–90 °C and then AFGO (0.1% *w*/*v*) was added in GG transparent solution by ultrasound for 1 h. The mineral oil (90% *w*/*w*) was slowly mixed with GG/AFGO (10% *w*/*w*) heated at 50 °C by water-in-oil emulsion using a 23G homogenizer (400 rpm) blender for 10 min. Then, Span 85 (0.5% *w*/*v*) and CaCl_2_ (10 mL, 1.25%) were added and kept stirring at 50 °C for 1 h. After that, CaCl_2_ (10 mL, 1.25%) was added again for 10 min. GG/AFGO microspheres were separated into different size fractions by sieving using 25-mesh (710 μm), 40-mesh (425 μm), 50-mesh (300 μm) and 70-mesh (212 μm) standard sieves. The microsphere used the acetone to wash three times and was re-dispersed in deionized water. In another experiment, the AFGO (0.1% *w*/*v*) was added to 2 mg Dox in 10 mL deionized water for 3 days. The synthesis of GG/AFGO–Dox microsphere was conducted by the emulsification process using the hydrophilic phase containing GG mixed AFGO–Dox solution (10% *w*/*w*) with the hydrophobic phase (mineral oil, 90% *w*/*w*) and all the other parameters remained the same, respectively.

### 2.4. Characterization of GO, AFGO, GG/AFGO Microsphere and GG/AFGO–Dox Microsphere

The zeta potential and chemical state of the GO (1 mg/mL in deionized water, room temperature) and AFGO (1 mg/mL in deionized water, room temperature) were analyzed by a zeta potential analyzer (Mastersizer 2000, Malvern, UK) and fourier transform infrared spectroscopy (FTIR) (Tensor 27). The particle size of the AFGO, GG microsphere and GG/AFGO–Dox microsphere was measured by a laser diffraction particle size analyzer (Coulter LS230 and Mastersizer 2000, Malvern, UK). The morphology of the GG/AFGO microsphere and GG/AFGO–Dox microsphere were characterized by SEM (Jeol JSM-6400, Tokyo, Japan). The elemental composition was performed by energy-dispersive X-ray spectroscopy (EDS).

### 2.5. Release and Kinetic Model of AFGO and DOX in GG/AFGO–Dox Microsphere

The standard curve of AFGO concentration was created by counting the number of AFGO in different concentrations using the counter, because the particle size of AFGO is concentrated. The amount AFGO and DOX released from different sizes of GG/AFGO and GG/AFGO–Dox microsphere was calculated in 15 mL centrifuge tubes. The GG/AFGO microsphere (1 mg/mL) was immersed in 5 mL of phosphate (1 M, pH 7.4) and incubated at 37 °C with shaking for 45 days. The sample was centrifuged at 300× *g* for 10 min and the supernatant was taken to calculate the concentration of AFGO every four days. In another experiment. The GG/AFGO–Dox microsphere (1 mg/mL) was immersed in 2 mL of phosphate (1 M, pH 7.4) and incubated at 37 °C with shaking for 45 days. The sample was centrifuged at 350× *g* for 10 min and the supernatant was taken to measure the Dox concentration by spectrophotometry at 480 nm every four days. Five kinetic models—Zero, First, Higuchi, Hixson–Crowell and Korsmeyer–Peppas—were used.

### 2.6. HepG2 Uptake of AFGO

Human hepatoma cells (HepG2) were used as the model cell line for evaluation of uptake of AFGO nanoparticles. Cells were obtained from the Bioresource Collection and Research Center (BCRC), Hsinchu, Taiwan. The cells were grown on EMEM containing 1 mM sodium pyruvate supplemented with 10% fetal bovine serum and 40 mg/mL gentamycin, at 37 °C. Cells were trypsinized on reaching 90% confluence. HepG2 cells were seeded on glass coverslips and allowed to adhere by incubating for a period of 24 h at 37 °C. The medium was discarded and replaced with AFGO nanoparticle medium (experimental group) and without AFGO nanoparticles medium (control). The glass coverslips were. then incubated at 37 °C for 3, 9 and 24 h. Then, we washed the glass coverslips with cold PBS three times to remove the excess nanoparticles not taken up by the cells and then fixed the cell with 1% glutaraldehyde. In order to remove residue nanoparticles completely, we used ultrasonic equipment to clean the glass coverslips again. The cells were viewed under the inverted fuorescence microscope (Zeiss Axi-oskop 2, Carl Zeiss Inc., Oberkochen, Germany).

### 2.7. In Vitro Cell Viability and Apoptosis Assay

HepG2 were seeded at 3 × 104 cells per well in the 96-well plate. The cells were incubated with various concentrations of Dox (0, 1.57, 2.35, 3.17 μM) and AFGO (0, 25, 50, 75 μg/mL) for 24 h. The cell viability was determined using the Presto reagent method according to the manufacturer’s protocol. In this study, the dose of Dox and AFGO were approximate to the cumulative release concentrations from GG/AFGO–Dox microspheres over 28 days. For cell apoptosis assays, HepG2 cells were seeded in 6-well plates at 4 × 10^5^ cells per well for 24 h. The cells were incubated with various concentrations of Dox and AFGO. After 24 h incubation, cells were harvested by trypsin-EDTA and incubated in 100 mL 1X buffer, followed by staining with FITC Annexin V (5 mL) and Propidium Iodide Staining Solution (5 mL) according to FITC Annexin V Apoptosis Detection Kit I with FITC Annexin V and Propidium Iodide Staining Solution. After incubation at room temperature for 15 min, samples were analyzed on a Flow Cytometer (FACSCalibur).

### 2.8. Vessel Embolization of Microsphere In Vivo

The New Zealand White rabbits were obtained from the Laboratory Animal Center (Taipei, Taiwai) at the weight of 2.6–3 kg. Before administration, the hairs of ear veins were removed. The Zoletil (Virbac)/Xylazine (20–40 mg/kg Z + 5–10 mg/kg X) was injected into the rabbit. Then, the GG/AFGO–DOX microsphere (20 mg/mL) was injected into the proximal part of the ear vein (0.15 mL/ear). After administration for 0, 4, 8, 12 days, the chemoembolization effect was carefully evaluated by color and shapes of rabbits’ ears. Ethics statement concerning animal work—the authors confirm that all experiments were performed in accordance with relevant guidelines and regulations. Animal work methods were carried out in accordance with procedures that were approved by the IACUC (Approval NO 2147: Vaild from 1 October 2019 to 31 July 2022) of the Chung-Shan Medical University Experimental Animal Center.

### 2.9. Statistical Analysis

All experiments were repeated at least 3 times and the Student’s test was performed to evaluate the difference in the average between groups. A value of * *p* < 0.05, ** *p* < 0.01 and *** *p* < 0.01 was taken as significant (Statistical Analysis: Microsoft Excel 2013).

## 3. Results and Discussion

### 3.1. Synthesis and Characterization of Amine-Functionalized Graphene Oxide (AFGO) and Amine-Functionalized Graphene Oxide–Doxorubicin (AFGO–Dox)

**Figure 1:**
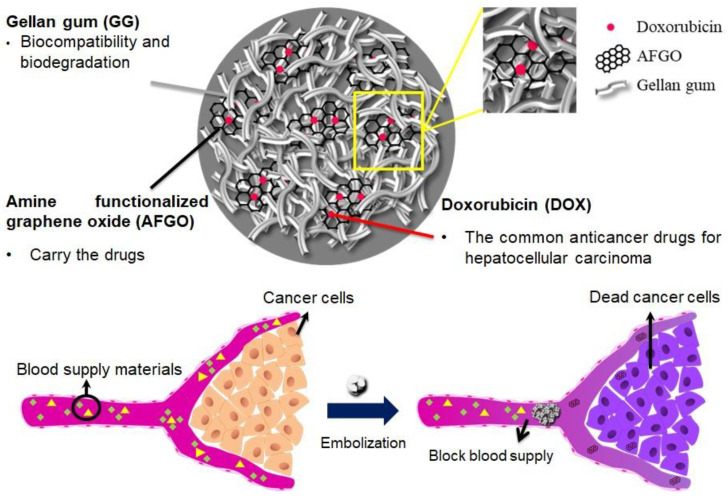
Scheme showing the embolic agent of microsphere-nanoparticle GG/AFGO–DOX blocking the blood supply and delivering AFGO–DOX in cancer treatment.

Dual-function microspheres that encapsulated a nanocarrier with an embolic agent (GG/AFGO–Dox) were developed for transcatheter arterial chemoembolization (TACE) to block nutrient delivery and enhance drug absorption, leading to tumor cell starvation and subsequent cell apoptosis (Figure 1). Many reports have highlighted the potential of graphene oxide (GO) to function as a drug carrier due to its potential π–π and hydrophobic interactions with drugs that have aromatic rings [27,28]. In addition, amine-functionalized graphene oxide (AFGO) was further modified to enhance drug hydrophilic interactions and to facilitate graphene assembly with the polymer. A GO size of between 0.5 and 25 μm enables cell membrane penetration, thereby increasing drug availability [29].

**Figure 2:**
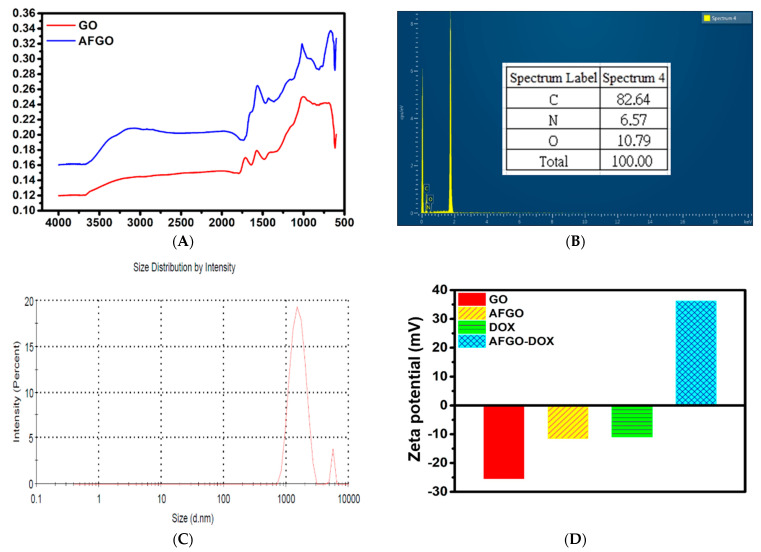
(**A**) FTIR spectrum of GO (red) and AFGO (blue). (**B**) EDS analysis of AFGO. (**C**) Size distribution of AFGO was evaluated using a laser diffraction particle size analyzer. (**D**) Zeta potentials of GO, AFGO, DOX and AFGO–DOX.

Following the modification of GO, the structure of AFGO was analyzed using FTIR spectroscopy and EDS to confirm it was successfully prepared. In accordance with Yao, S.Z. et al. (2013) [30], as shown in Figure 2A, the peak at 1712 cm^−1^ was replaced with a new peak at 1163 cm^−1^, representing C–N binding [31]. The amine group produced a peak at 667 cm^−1^. Moreover, the amount of nitrogen in AFGO was 6.57% and the size of AFGO was 1.564 ± 0.414 μm, as shown in Figure 2B,C. In addition, the zeta potentials of GO and AFGO were −25.4 and −11.5 mV, respectively, as shown in Figure 2D. The negative zeta potential of GO was supposedly due to the negatively charged surface of the GO sheet, which is attributed to the presence of carboxyl groups, whereas the relatively more positive zeta potential of the AFGO nanosheets was attributed to the positive charges that were induced by the presence of the NH_2_ groups. Additionally, AFGO that is dispersed in deionised water (pH 5.5) presents a weak negative charge. According to Fang et al. [32], the zeta potential of AFGO varies depending on the pH value, being positive at pH < 4, and negative at pH > 4. To enhance the therapeutic efficiency of Dox, it was conjugated to AFGO via electrostatic and π–π interactions (AFGO–Dox complex). As shown in Figure 2D, the zeta potentials of Dox–HCl and AFGO–Dox were −10.9 mV and +36.2 mV, respectively. The DOX–HCl (negative) is a product of the interaction between an acid and a base, whereby doxorubicin acts as a weak base and HCl is an acid. Upon mixing Dox–HCl and AFGO in water, HCl dissociates into H^+^ and Cl^−^ and the pH decreases (pH 4.0). In addition, AFGO–Dox complex with the former leading to the protonation of N in doxorubicin, thus establishing a positive charge. The AFGO–Dox complex exhibited a positive charge also due to the presence of the amine group, thus confirming that Dox was chemical bonder with the AFGO nanoparticle. These results illustrate the potential for successfully synthesized AFGO–Dox to penetrate the cell membrane and act as a drug carrier through electrostatic interactions.

### 3.2. Synthesis and Characterization of GG and GG/AFGO–Dox Microspheres

**Figure 3:**
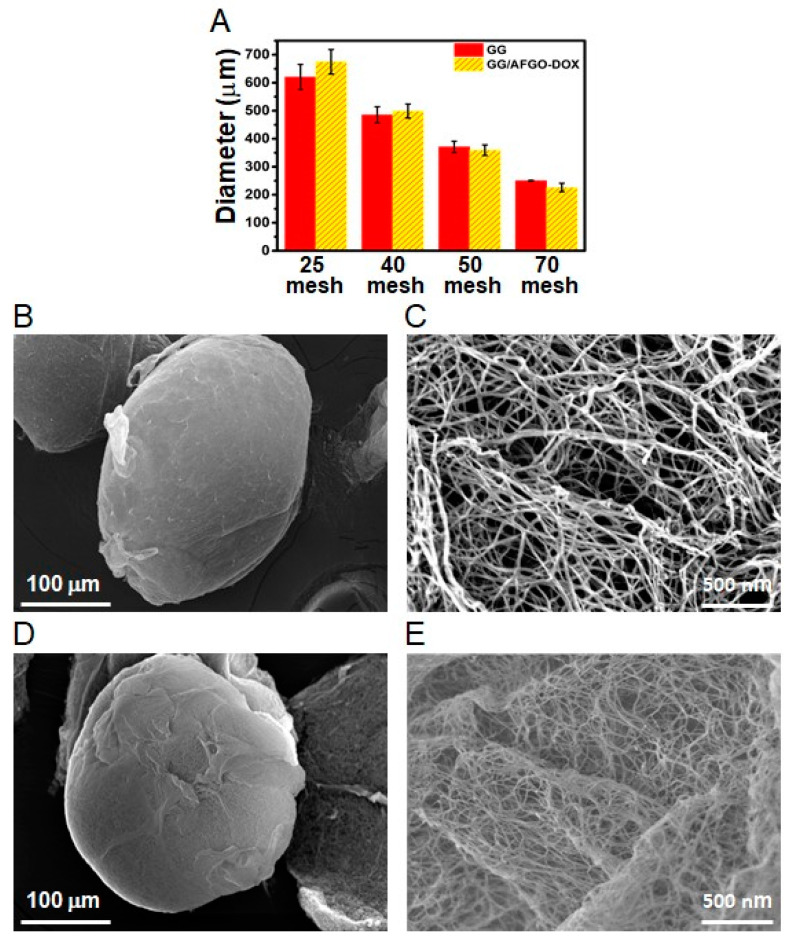
(**A**) Size distribution of GG and GG/AFGO–Dox following separation using 25, 40, 50, and 70 mesh sieves, and their evaluation using a laser diffraction particle size analyzer. SEM images of GG (**B**) surface and (**C**) inner microsphere. SEM images of GG/AFGO–DOX (**D**) surface and (**E**) inner microsphere.

For the TACE procedure, blocking the blood supply closest to the tumor site is important for reducing the damage to normal cells. Therefore, it is essential that the embolic microsphere has a suitable diameter for TACE, which is based on blood vessel size. The size of microspheres used for TACE in clinical trials has ranged from 50 to 500 μm [33]. To evaluate the size variation in the GG and GG/AFGO–Dox microspheres, their diameter was measured using a laser diffraction particle size analyzer. A mixture of GG and GG/AFGO–Dox microspheres was filtered using 25, 40, 50 and 70 mesh sieves, thereby separating the microspheres on the basis of diameter. As shown in Figure 3A, the 25-mesh sieve was able to separate GG (620 ± 44 μm) and GG/AFGO–Dox (674 ± 43 μm) microspheres that were over 500 µm in size, where-as the 40, 50 and 70 mesh sieves yielded GG microspheres of 485 ± 28, 371 ± 20 and 250 ± 17 μm and GG/AFGO–Dox microspheres of 498 ± 25, 359 ± 19 and 226 ± 15 μm, respectively. Following the addition of AFGO, the sizes of the GG/AFGO–Dox and GG microspheres were similar. To confirm the encapsulation of AFGO–Dox, we analyzed the morphology of the microspheres using SEM. The SEM images in Figure 3B, C show the elliptical morphology and smooth and compact surface of the GG microspheres. This round shape is very important to allow an effective ischemic effect for TACE of cancer. Furthermore, the internal structure of the GG particles can clearly be seen as a loose and fibrous structure. On the other hand, the GG/AFGO–Dox microspheres were spherical, with a network structure on their surface, as shown in Figure 3D. Moreover, the internal structure of the GG/AFGO–Dox particles was tight, as shown in Figure 3E. This dense structure was formed by the entanglement of the GG and AFGO sheets. These results confirmed the free and AFGO–Dox-loaded gellan gum microcapsules were not changed for their size distribution and morphology.

### 3.3. AFGO and Dox Release and Kinetic Model in GG/AFGO–Dox Microspheres

**Figure 4:**
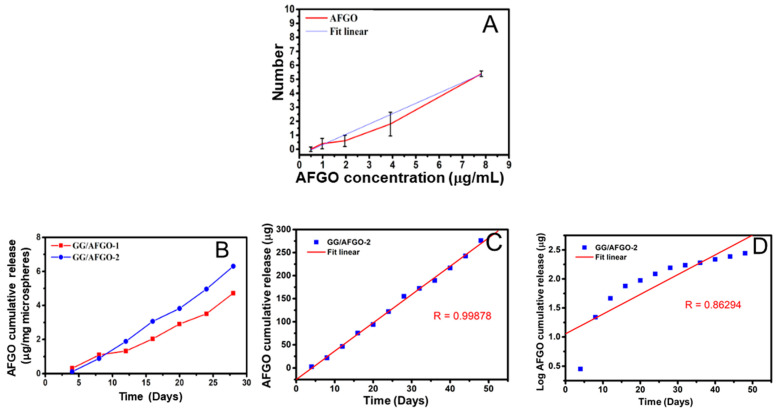

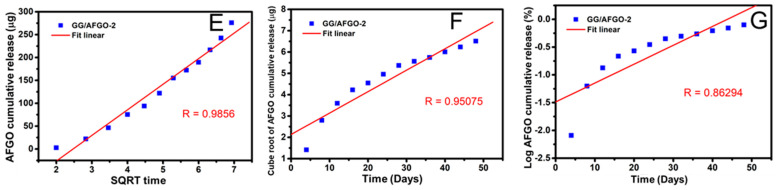
(**A**) Profile of AFGO release (**B**) Profile of AFGO release from GG/AFGO–Dox-1 (674 ± 43 μm) and GG/AFGO–Dox-2 (226 ± 15 μm) over 28 days. AFGO release according to the following kinetic models: (**C**) zero, (**D**) first, (**E**) Higuchi, (**F**) Hixson–Crowell and (**G**) Korsmeyer–Peppas.

Many studies have determined the concentration of AFGO using a spectrophotometer; however, AFGO was not water-soluble in this study [34,35]. Therefore, this study determined the concentration of AFGO by using a microscope to count the number of molecules. As shown in Figure 4A, the *R* value of the standard curve was credible (0.99). To improve the efficiency of AFGO–Dox cancer cell entry, the release and kinetic mechanisms of AFGO encapsulated in the large (GG/AFGO–Dox-1 (674 ± 43 μm)) and small (GG/AFGO–Dox-2 (226 ± 15 μm)) microspheres were analyzed. As shown in Figure 4B, the cumulative release concentrations of GG/AFGO–Dox-1 and GG/AFGO–Dox-2 over 28 days were 4 and 6 μg/mg microspheres, respectively. The higher release concentration of the latter was due to its greater number of microspheres per unit weight. However, their release slopes were similar due to the structures of the microspheres being identical. Moreover, their release over 14 to 28 days was faster than their release over 0 to 14 days because of the slow degradation of GG.

Five drug release kinetic models were used to evaluate the AFGO release from the GG/AFGO–Dox-2 microspheres: zero-order, first-order, Higuchi, Hixson–Crowell and Korsmeyer–Peppas [36,37]. The *R* values that were obtained for each model were 0.9988, 0.8629, 0.9856, 0.9508 and 0.8629, respectively, as shown in Figure 4C–G. The best fits were obtained using the zero-order and Higuchi models. The former suggested that the GG/AFGO–Dox-2 microspheres were not degraded and AFGO was released slowly and steadily, whereas the latter suggested that the GG/AFGO–Dox-2 microspheres were degraded over time, leading to a loose structure, thereby facilitating the penetration of AFGO into GG. Considering that GG is a slowly degrading material, the kinetics of AFGO release from the GG/AFGO–Dox-2 microspheres was best described by the Higuchi model. Furthermore, the introduction of AFGO improved the stability of the gellan gum particles, rendering them akin to a steel ball, thereby also satisfying the zero-order model. Overall, these results indicate that the GG/AFGO–Dox microspheres roughly exist in the form of steel balls, which prevents the substantial release of AFGO in a short period of time while simultaneously achieving embolization and chemotherapy. Furthermore, the embolization is only temporary due to the slow degradation of the embolic agent.

**Figure 5:**
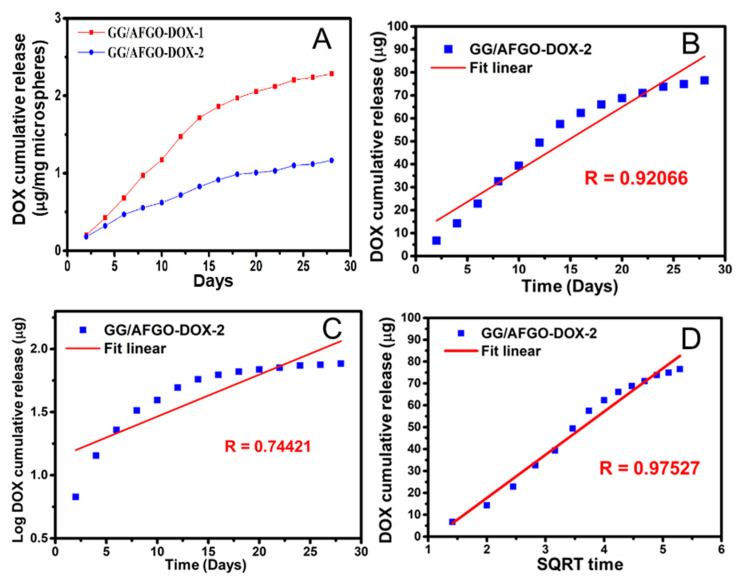

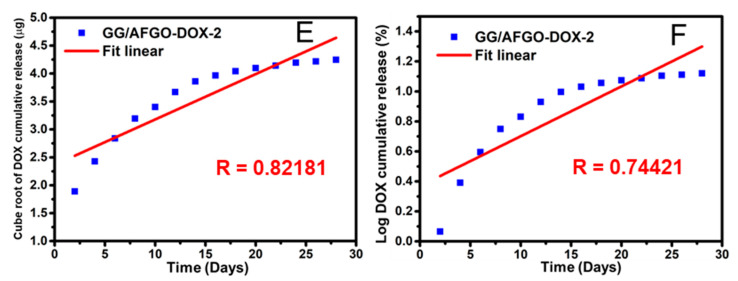
(**A**) Profile of Dox release from the GG/AFGO–Dox-1 (674 ± 43 μm) and GG/AFGO–Dox-2 (226 ± 15 μm) over 28 days. Dox release according to the following kinetic models: (**B**) zero, (**C**) first, (**D**) Higuchi, (**E**) Hixson–Crowell, and (**F**) Korsmeyer–Peppas.

We sought to determine the effect of the microsphere diameter on Dox release using kinetic models. The cumulative release concentrations of Dox from GG/AFGO–Dox-1 and GG/AFGO–Dox-2 were 2.31 and 1.18 μg/mg microspheres, respectively, over 28 days, as shown in Figure 5A. The difference in concentration was due to the latter having a larger surface area per unit weight. Once again, five kinetic models were used to analyze Dox release from the GG/AFGO–Dox-2 microspheres: zero-order, first-order, Higuchi, Hixson–Crowell and Korsmeyer–Peppas. The *R* values obtained for each model were 0.9302, 0.8054, 0.9770, 0.9314 and 0.8054, respectively, as shown in Figure 5B–E. The drug release results best conformed to the Higuchi model, in which drug release is characterized by proceeding through diffusion, a structure with varying geometric shapes or matrix characteristics (e.g., porosity), and a release rate that is dependent on the material thickness [38]. These characteristics indicate that Dox was initially released from the surface and then from the inner layer, thus achieving slow release through continuous diffusion. The kinetic models of AFGO and Dox release were consistent with their encapsulation.

### 3.4. Verification of the Cellular Uptake of AFGO

**Figure 6:**
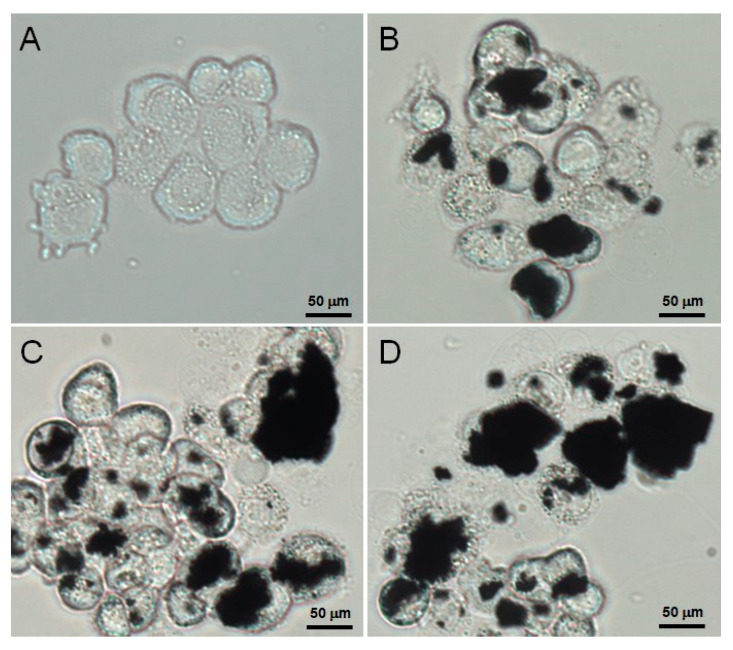
Uptake of AFGO by HepG2 cells observed with a microscope at (**A**) 0 h, (**B**) 3 h, (**C**) 9 h and (**D**) 24 h (scale bar = 50 μm; 400×).

The particle size analysis of AFGO (Figure 2C) revealed that the size of graphene should enable cell membrane penetration. To verify this possibility, a time course of AFGO absorption by the HepG2 cells was investigated through microscopy analysis at 3, 9 and 24 h, as shown in Figure 6. The level of AFGO that was phagocytosed into the cell increased with time, thus showing that AFGO was able to penetrate into the HepG2 cells.

### 3.5. Cell Viability and Apoptosis of GG/AFGO–Dox

**Figure 7:**
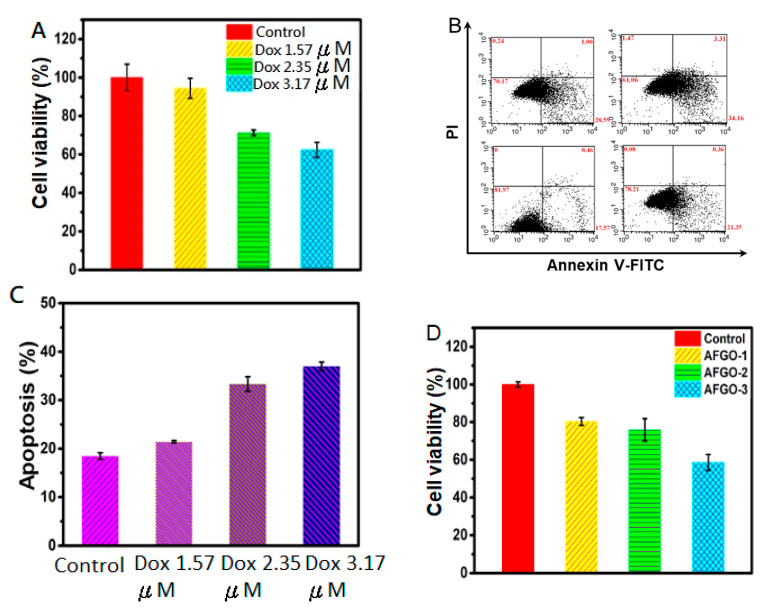

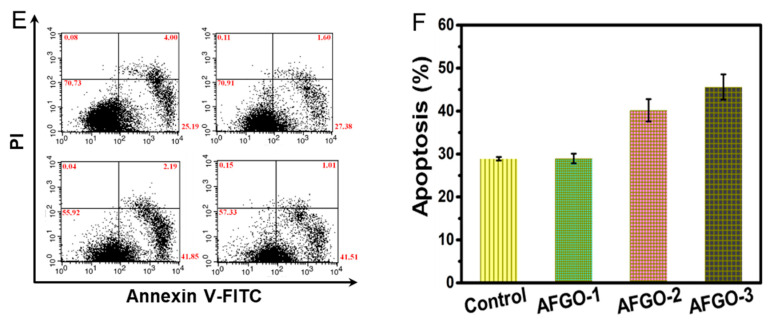
Analysis of cell viability and apoptosis by flow cytometry. (**A**) Cell viability of HepG2 cells after 24 h incubation in medium with 0 (control), 1.57, 2.35, 3.17 μM of DOX. (**B**,**C**) Apoptosis induced in HepG2 cells treated with different concentrations of DOX for 24 h by flow cytometry. (**D**) Cell viability of HepG2 cells after 24 h incubation in a medium with AFGO-1 (25 μg/mL), AFGO-2 (50 μg/mL), AFGO-3 (75 μg/mL) and without AFGO (control). (**E**,**F**) Apoptosis induced in HepG2 cells treated with AFGO-1 (25 μg/mL), AFGO-2 (50 μg/mL) and AFGO-3 (75 μg/mL) for 24 h by flow cytometry. Two-tailed Student’s test was used.

Embolic agents mainly block the blood supply, resulting in starvation; however, they do not react directly with the tumor cells. Therefore, we sought to test the cytotoxicity of DOX and AFGO on HepG2 cells by the Presto reagent method and it was found that Dox and AFGO were toxic to HepG2 cells dose-dependently. The IC50 of doxorubicin in HepG2 cell is 1.1 μM/mL [22]. The experimental results showed that the amount of released doxorubicin from 226, 359 and 498 μm GG/AFGO–DOX microspheres was approximately 1.4-, 2.1- and 2.9-fold above the IC50 value. On the basis of the results observed, we assert that three sizes of multifunctional GG/AFGO–DOX microspheres prepared in this study meet the requirements of TACE applications. In addition, graphene oxide particles with ammonia modification (AFGO), tend to aggregate into cell culture which influences their effective size and may affect their induced cytotoxicity [39]. Therefore, at higher concentrations of AFGO, aggregation was much more pronounced and the toxicity increased. Moreover, we examined whether Dox induced apoptosis using the FITC Annexin V Apoptosis Detection Kit I. As shown in Figure 7B, the apoptosis rates were 17.57%, 21.35%, 28.59% and 34.16% for the control, Dox 1.57, 2.35, 3.17 μM treatments, respectively. Different concentrations of AFGO treatments induced significant apoptosis compared with the control, as shown in Figure 7C. Therefore, AFGO was able to increase the cytotoxic effect of Dox, in addition to inducing cell death. Park et al. [40] found that graphene oxide, reduced graphene oxide and sodium lauryl sulfate graphene oxide at concentrations of 3–25 μg/mL induced mild cytotoxicity of A549 cells, whereas concentrations of 50–400 μg/mL induced severe cytotoxicity [41,42]. To illustrate the cytotoxicity of AFGO, different concentrations were investigated in vitro. As shown in Figure 7D, the cell viabilities were reduced to 19%, 22% and 39% following treatment with 25 (AFGO-1), 50 (AFGO-2) and 75 (AFGO-3) μg/mL, respectively. All concentrations exhibited significant cytotoxicity compared to the control group, in line with the previous report, although the cell line used and the graphene modification were different. Furthermore, the level of induced apoptosis was used to evaluate the effect of different concentrations of AFGO on liver cancer cells. As shown in Figure 7E, the apoptosis rates were 25.19%, 27.38%, 41.85% and 41.51% for the control, AFGO-1, AFGO-2 and AFGO-3 reactions, respectively. All three concentrations of AFGO induced significant apoptosis compared with the control, as shown in Figure 7F. These results indicate that AFGO was toxic toward HepG2 cells at concentrations over 25 μg/mL.

### 3.6. Vessel Embolization of GG/AFGO In Vivo

**Figure 8:**
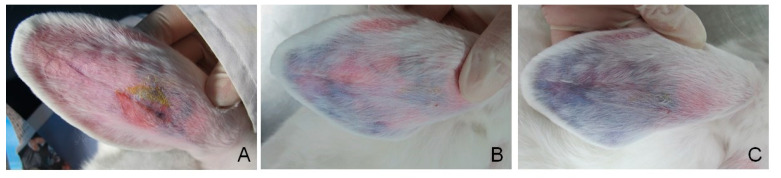
The embolization of GG/AFGO–Dox microspheres using the macroscopic view observed at (**A**) day 0, (**B**) day 6 and (**C**) day 12.

To evaluate the embolization efficiency of GG/AFGO–Dox (226 μm) in vivo, we used a rabbit ear model that had been established in our previous study [22]. As shown in Figure 8, following the injection of GG/AFGO–Dox microspheres, the central auricular artery and its branches were visible in the ear (Figure 8A, day 0). Six days after embolization (Figure 8B), the blood flow at the tip of the ear was blocked. In addition, there was no obvious edema or inflammation, thus confirming the good biocompatibility of GG/AFGO–Dox microspheres. Twelve days after embolization (Figure 8C), the branches of the artery between the injection site and the tip of the ear were occluded, resulting in ischemic necrosis. These results indicate that the GG/AFGO–Dox microspheres successfully achieved embolization.

## 4. Conclusions

In conclusion, embolization agents based on gellan gum polymer were developed. The properties of an embolization agent play an important role in guiding clinical choices. In this study, we used gellan gum to encapsulate a nanosized (AFGO) drug delivery system, yielding GG/AFGO–Dox microspheres of different sizes as a multifunctional embolic agent. The gellan gum-based microspheres are suitable for long-acting vascular embolization with excellent biocompatibility. Furthermore, they effectively improved the stability of doxorubicin, strengthened its affinity toward cancer cells, and increased its availability. In the future, our aim is to provide better embolization agents for the treatment of liver or other cancers using this device.

## Data Availability

The data presented in this study are available on request from the corresponding author.
